# Complete host specificity test plant list and associated data to assess host specificity of *Archanara geminipuncta* and *Archanara neurica*, two potential biocontrol agents for invasive *Phragmites australis* in North America

**DOI:** 10.1016/j.dib.2018.06.068

**Published:** 2018-06-26

**Authors:** Bernd Blossey, Patrick Häfliger, Lisa Tewksbury, Andrea Dávalos, Richard Casagrande

**Affiliations:** a Department of Natural Resources, Fernow Hall, Cornell University, Ithaca, NY 14853, USA; b CABI, Rue des Grillons 1, CH-2800 Delémont, Switzerland; c Department of Plant Sciences and Entomology, University of Rhode Island, Kingston, RI 02881, USA; d Biological Sciences, SUNY Cortland, 1215 Bowers Hall, Cortland, NY 13045, USA

**Keywords:** *Archanara*, Biological weed control, Host specificity, *Phragmites*, Sub-species level specificity, Wetlands

## Abstract

Introduced European genotypes of *Phragmites australis* are invasive and widespread in North America. Decades of management using herbicide and other means have failed to control the species and its range and populations continue to expand. Allowing continued invasion threatens native wetland biota and an endemic North American subspecies *Phragmites australis americanus*. The lack of conventional management to control introduced *P. australis* triggered research to assess host specificity of two European noctuid moths, *Archanara geminipuncta* and *Archanara neurica*. These two species are considered particularly promising potential biocontrol agents for introduced *P. australis*. Here we provide the complete and approved list of test plants used to assess host specificity of *A. geminipuncta* and *A. neurica*. This includes data on neonate larval acceptance and survival under no-choice conditions, and oviposition tests for all plant species tested, including for different *Phragmites* subspecies currently occurring in North America. We further provide temperature profiles of select cities in the temperate native European distribution of the two noctuids and those in southern US climates. We used these long-term temperature records to assess whether overwintering eggs of *A. geminipuncta* and *A. neurica* can survive under climate conditions typical for the Gulf Coast region in North America. This data article refers to “Host specificity and risk assessment of *Archanarageminipuncta* and *Archanaraneurica*, two potential biocontrol agents for invasive *Phragmitesaustralis* in North America Biol. Control (2018)”.

**Specifications Table**

**Table:** 

Subject area	*Biology, plant insect interactions*
More specific subject area	*Invasive plants, biological weed control*
Type of data	*Tables of test plants, results of oviposition and larval development tests, and temperature profiles*
How data was acquired	*Reference literature on plant taxonomy, USDA Plants Database*[1] and city data available on the web:
	http://www.usclimatedata.com
	https://www.timeanddate.com
Data format	*Raw*
Experimental factors	*None*
Experimental features	*We used selected plant species to test acceptance or suitability for larval development or oviposition by two noctuid moths.*
	*We use temperature profiles of select locations to program incubators to resemble local climate conditions to test for winter survival of noctuid eggs.*
Data source location	[1]
	http://www.usclimatedata.com
	https://www.timeanddate.com
Data accessibility	*With this article*

**Value of the data**

• We provide a comprehensive list and results for all test plant species used to assess host specificity (oviposition and larval development) for *Archanara geminipuncta* and *Archanara neurica*. We provide data on larval survival, which provides a full overview of the selectivity of these moth species, and hence the safety of other wetland plants.
• We provide data on temperature profiles we used to assess the possibility of winter survival under different climate conditions for select locations in Europe and North America. This allows an assessment of the ability of *A. geminipuncta* and *A. neurica* to colonize regions with different climate conditions in North America based on their current distribution in Europe.

## 1 Data

The approval of specialized herbivores as biological weed control agents in North America requires extensive host specificity testing and federal (US and Canada) review [2]. Procedures to select appropriate test plant species are largely standardized and our host-plant selection was based on plant phylogeny, species of conservation or agricultural concerns, plants that are attacked by congeners of *A. geminipuncta* and *A. neurica*, and chemically similar plants [2], [3]. In determining appropriate plant species for host-range testing, we used information provided in the USDA PLANTS database [1] that included taxonomic relationships, distribution in North America, and status as native or introduced. The selection of plant species (Table 1) was approved by the Technical Advisory Group for Biological Control Agents of Weeds (TAG) in 2009.

**Table 1:** TAG Category^1^, plant species used to determine host range of *Archanara geminipuncta* and *Archanara neurica*, potential biocontrol agents of invasive *Phragmites australis*, initiation of larval feeding or survival (AG indicates *A geminipuncta*, AN indicates *A. neurica*) and plant distribution according to USDA Plants Database in North America [1]. Initiation of larval feeding would result in follow-up tests [6]. (For two letter US State, or Canadian Province abbreviations please consult https://pe.usps.com/text/pub28/28apb.htm, or www.comeexplorecanada.com/abbreviations.php.

**TAG**	**Species**	**Larval Feeding**	**Distribution in North America**	
**Cat.**^**A**^				
			**US**	**Canada**
**Poaceae, Arundinoideae**				
Target	*Phragmites australis* (Cav.) Trin. ex Steud., Haplotype M	AG/AN	CA, CT, DE, DC, HI, ID, IL, IN, IA, LA, ME, MD, MA, MN, NE, NV, NH, NJ, NY, NC, OH, OR, PA, RI, SC, TX, UT, VT, VA, WA, WI, WY	AB, BC, MB, NB, NL, NS, ON, QC, SK
1*	*P. australis americanus* Saltonstall, P.M. Peterson & Soreng, (ME haplotypeE)	AG/AN	Arrowsic, ME	
1	*P. australis americanus* (NB haplotype S)	AG/AN		Moncton, NB
1	*P. australis americanus* (RI haplotype AB)	AG/AN	Block Island, RI	
1	*P. australis americanus* (NY haplotype E)	AG/AN	Montezuma NWR, NY	
1	*P. australis americanus* (DE haplotype F)	AG/AN	Dover, DE	
1	*P. australis berlandieri* (E. Fourn.) C.F. Reed, (Zellwood, FL haplotype I)	AG/AN	AZ, AL, FL, LA, MS, NM, TX	
3d*	*Arundo donax* L.	AG	AL, AZ, AR, CA, DE, DC, FL, GA, IL, KS, KY, LA, MD, MS, MO, NV, NM, NC, OK, SC, TN, TX, UT, VA, WV	
3d*	*Cortaderia selloana* (Schult. & Schult.f.) Asch. & Graebn.	AG	AL, CA, GA, LA, NJ, OR, SC, TN, TX, UT, VA, WA,	
**Poaceae, Aristoideae**				
3d	*Aristida purpurea* Nutt.	–	AZ, AR, CA, CO, ID, IL, IA, KS, LA, MN, MT, NE, NV, NM, NC, ND, OK, OR, SC, SD, TX, UT, VT, WA, WY	AB, BC, MB, SK
**Poaceae, Chloridoideae**				
3d	*Distichlis spicata* (L.) Greene	–	AL, AZ, CA, CO, CT, DE, DC, FL, GA, HI, ID, IL, IA, KS, LA, ME, MD, MA, MN, MS, MO, MT, NE, NV, NH, NJ, NM, NY, NC, ND, OH, OK, OR, PA, RI, SC, SD, TX, UT, VA, WA, WI, WY	AB, BC, MB, NB, NS, NT, ON, PE, SK
**Poaceae, Chloridoideae**				
3d, 7	*Spartina alterniflora* Loisel.	AG/AN	AL, CA, CT, DE, DC, FL, GA, LA, ME, MD, MA, MS, NH, NJ, NY, NC, OR, RI, SC, TX, VA, WA	NB, NL, NS, PE, QC
3d, 4, 7	*Spartina cynosuroides* L. (Roth)	AG	AL, CT, DE, FL, GA, LA, MD, MA, MS, NJ, NY, NC, PA, RI, SC, TX, VA	
3d, 7	*Spartina pectinatus* Bosc ex Link	–	AZ, CO, DC, DE, ID, IL, IN, IA, KS, KY, LA, MA, ME, MI, MN, MO, MT, NC, NE, NH, NM, NJ, NY, ND, OH, OK, OR, PA, RI. SC, SD, TN, TX, UT, VT, VA, WA, WI, WV, WY	AB, BC, MB, NB, NL, NS, NT, ON, PE, QC, SK, YT
3d	*Muhlenbergia racemosa* Michx.	–	AZ, CO, DC, ID, IL, IN, IA, KS, KY, ME, MI, MN, MO, MT, NE, NV, NH, NM, NY, ND, OK, OR, SD, TX, UT, VT, VA, WA, WI, WY	AB, BC, MB, NS, ON, QC, SK
3	*Eragrostis trichodes* (Nutt.)	AN	AL, AR, CO, DC, IL, IN, IA, KS, LA, MI, MN, MS, MO, NE, NM, NY, OH, OK, SD, TN, TX, VA, WI, WY	
**Poaceae, Danthonioideae**				
3d	*Danthonia spicata* (L.)	–	AL, AK, AZ, AR, CO, CT, DE, DC, FL, GA, ID, IL, IN, IA, KS, KY, LA, ME, MD, MA, MI, MN, MS, MO, MT, NE, NH, NJ, NM, NY, NC, ND, OH, OK, OR, PA, RI, SC, SD, TN, TX, VT, VA, WA, WV, WI, WY	AB, BC, MB, NB, NL, NS, NT, ON, PE, QC, SK, YT
**Poaceae, Ehrhartoideae**				
3d	*Leersia oryzoides* (L.)	–	AL, AZ, AR, CA CO, CT, DE, DC, FL, GA, ID, IL, IN, IA, KS, KY, LA, ME, MD, MA, MI, MN, MS, MO, MT, NE, NV, NH, NJ, NM, NY, NC, ND, OH, OK, OR, PA, RI, SC, SD, TN, TX, UT, VT, VA, WA, WV, WI, WY	BC, MB, NB, NS, ON, PE, QC, SK,
3d*	*Oryza sativa* L	AG	AL, AR, CA, GA, IL, LA, MI, MO, NC, OK, SC, TN, TX, VA	
3d, 6	*Zizania aquatica* L.	AN	AL, CT, DE, DC, FL, GA, IL, IN, IA, LA, ME, MD, MA, MI, MN, MS, NH, NJ, NY, NC, OH, PA, RI, SC, VT, VA, WI	AB, BC, MB, NB, NL, ON, QC
3d,4,6	*Zizaniopsis miliacea (*Michx.)	–	AL, AR, FL, GA, IL, KY, LA, MD, MI, MO, NC, OK, SC, TN, TX, VA	
**Poaceae, Panicoideae**				
3d,6*	*Sorghum bicolor* (L.)	–	AL, AZ, AR, CA CO, CT, DC, FL, GA, ID, IL, IN, IA, KS, KY, LA, ME, MD, MA, MI, MN, MS, MO, MT, NE, NV, NH, NJ, NM, NY, NC, ND, OH, OK, OR, PA, RI, SC, SD, TN, TX, UT, VT, VA, WA, WI, WY	ON, QC
3d	*Andropogon gerardii* Vitman	–	AL, AZ, AR, CO, CT, DE, DC, FL, GA, IL, IN, IA, KS, KY, LA, ME, MD, MA, MI, MN, MS, MO, MT, NE, NH, NJ, NM, NY, NC, ND, OH, OK, PA, RI, SC, SD, TN, TX, UT, VT, VA, WV, WI, WY	MB, ON, QC, SK
3d, 4	*Sorghastrum nutans* (L.)	–	AL, AZ, AR, CO, CT, DE, DC, FL, GA, IL, IN, IA, KS, KY, LA, ME, MD, MA, MI, MN, MS, MO, MT, NE, NH, NJ, NM, NY, NC, ND, OH, OK, PA, RI, SC, SD, TN, TX, UT, VT, VA, WV, WI, WY	MB, ON, QC, SK
3d, 4	*Tripsacum dactyloides* (L.)	–	AL, AK, AR, CT, DE, DC, FL, GA, IL, IN, IA, KS, KY, LA, MD, MA, MI, MS, MO, NE, NJ, NY, NC, OH, OK, PA, RI, SC, TN, TX, VA, WV	
3d*	*Saccharum officinarum* L.	AN	AL, FL, LA, MI, TX	
3d,6*	*Zea mays* L.	–	AL, AZ, AR, CA CO, CT, DC, FL, GA, ID, IL, IN, IA, KS, KY, LA, ME, MD, MA, MI, MN, MS, MO, MT, NE, NV, NH, NJ, NM, NY, NC, OH, OR, PA, RI, SC, TN, TX, UT, VT, VA, WA, WV, WI, WY	ON, QC
3d	*Panicum virgatum* L.	–	AL, AZ, AR, CO, CT, DE, DC, FL, GA, ID, IL, IN, IA, KS, KY, LA, ME, MD, MA, MI, MN, MS, MO, MT, NE, NV, NH, NJ, NM, NY, NC, ND, OH, OK, PA, RI, SC, SD, TN, TX, UT, VT, VA, WV, WI, WY	MB, NS, ON, QC, SK
3d*	*Setaria italica* (L.)	–	AL, AZ, AR, CA CO, CT, DE, DC, FL, GA, IL, IN, IA, KS, KY, LA, ME, MD, MA, MI, MN, MS, MO, MT, NE, NH, NJ, NM, NY, NC, ND, OH, OK, OR, PA, RI, SC, SD, TN, TX, VT, VA, WA, WV, WI, WY	AB, BC, MB, NB, NS, ON, QC
**Poaceae, Pooideae**				
3d*	*Lolium perenne* L.	–	AL, AK, AZ, AR, CA CO, CT, DE, DC, FL, GA, ID, IL, IN, IA, KS, KY, LA, ME, MD, MA, MI, MN, MS, MO, MT, NE, NV, NH, NJ, NM, NY, NC, ND, OH, OK, OR, PA, RI, SC, SD, TN, TX, UT, VT, VA, WA, WV, WI, WY	AB, BC, MB, NB, NL, NS, NT, ON, PE, QC, SK, YT
3d*	*Dactylis glomerata* L.	–	AL, AK, AZ, AR, CA CO, CT, DE, DC, FL, GA, ID, IL, IN, IA, KS, KY, LA, ME, MD, MA, MI, MN, MS, MO, MT, NE, NV, NH, NJ, NM, NY, NC, ND, OH, OK, OR, PA, RI, SC, SD, TN, TX, UT, VT, VA, WA, WV, WI, WY	AB, BC, MB, NB, NL, NS, ON, PE, QC, YT
3d*	*Avena sativa* L.	–	AL, AK, AZ, AR, CA CO, CT, DE, DC, FL, GA, ID, IL, IN, IA, KS, KY, LA, ME, MD, MA, MI, MN, MS, MO, MT, NE, NV, NH, NJ, NM, NY, NC, ND, OH, OK, OR, PA, RI, SC, SD, TN, TX, UT, VT, VA, WA, WV, WI, WY	AB, BC, MB, NB, NL, NS, NT, ON, PE, QC, SK, YT
3d*	*Phalaris arundinacea* L.	AN	AL, AK, AZ, AR, CA CO, CT, DE, DC, ID, IL, IN, IA, KS, KY, ME, MD, MA, MN, MS, MO, MT, NE, NV, NH, NJ, NM, NY, NC, ND, OH, OK, OR, PA, RI, SD, TN, UT, VT, VA, WA, WV, WI, WY	AB, BC, MB, NB, NL, NS, NT, ON, PE, QC, SK, YT
3d	*Glyceria striata* (Lam.)	AN	AL, AK, AZ, AR, CA CO, CT, DE, DC, FL, GA, ID, IL, IN, IA, KS, KY, LA, ME, MD, MA, MI, MN, MS, MO, MT, NE, NV, NH, NJ, NM, NY, NC, ND, OH, OK, OR, PA, RI, SC, SD, TN, TX, UT, VT, VA, WA, WV, WI, WY	AB, BC, MB, NB, NL, NS, NT, ON, PE, QC, SK, YT
3d	*Elymus virginicus* L.	–	AL, AZ, AR, CT, DE, DC, FL, GA, IL, IN, IA, KS, KY, LA, ME, MD, MA, MI, MN, MS, MO, NE, NH, NJ, NM, NY, NC, ND, OH, OK, PA, RI, SC, SD, TN, TX, VT, VA, WV, WI, WY	AB, BC, MB, NB, NL, NS, ON, PE, QC, SK
3d,6*	*Hordeum vulgare* L.	–	AL, AK, AZ, AR, CA CO, CT, DC, FL, ID, IL, IN, IA, KS, KY, LA, ME, MD, MA, MI, MN, MS, MO, MT, NE, NV, NH, NJ, NM, NY, NC, ND, OH, OK, OR, PA, RI, SC, SD, TN, TX, UT, VT, VA, WA, WI, WY	AB, BC, MB, NB, NL, NS, NT, ON, PE, QC, SK, YT
3d,6*	*Secale cerelae* L.	–	AL, AK, AZ, AR, CA CO, CT, DC, FL, GA, ID, IL, IN, IA, KS, KY, LA, ME, MD, MA, MI, MN, MS, MO, MT, NE, NV, NH, NJ, NM, NY, NC, ND, OH, OR, PA, RI, SC, SD, TN, TX, UT, VT, VA, WA, WI, WY	AB, BC, MB, NB, NL, NS, NT, ON, PE, QC, SK, YT
3d,6*	*Triticum aestivum* L.	AG	AL, AK, AZ, AR, CA, CO, CT, DE, FL, GA, ID, IL, IN, IA, KS, KY, LA, ME, MD, MA, MI, MN, MS, MO, MT, NE, NV, NH, NJ, NM, NY, NC, OH, OK, OR, PA, RI, SC, SD, TN, TX, UT, VT, VA, WA, WV, WI, WY	AB, BC, MB, NB, NL, NS, NT, ON, TE, QC, SK, YT
3d*	*Agropyron cristatum* (L.)	–	AK, CA, CO, CT, DE, ID, IL, IN, IA, KS, KY, MA, MI, MN, MT, NE, NV, NH, NJ, NM, NY, ND, OK, OR, RI, SD, TX, UT, VT, WA, WY	AB, BC, MB, NL, NS, NT, ON, QC, SK, YT
**Poaceae, Bambusoideae**				
3d	*Arundinaria tecta* (Walter)	AG	AL, AR, FL, GA, LA, MD, MS, NC, NY, NJ, OK, PA, SC, TN, TX, VA	AB, BC, MB, NB, NF, NS, ON, PQ, SK
**Cyperaceae, Caricoideae**				
5	*Carex lurida* Wahlenb.	–	AL, AR, CT, DE, FL, GA, IL, IN, IA, KY, LA, ME, MD, MA, MI, MN, MS, MO, NH, NJ, NY, NC, OH, OK, PA, RI, SC, TN, TX, VT, VA, WV, WI	NB, NL, NS, ON, PE, QC
5	*Cyperus haspan L.*	–	AL, AR, FL, GA, LA, MS, NC, SC, TN, TX,	
**Cyperaceae, Cyperoideae**				
5, 7	*Schoenoplectus americanus* (Pers.)	AG	AL, AK, AZ, CA, CO, CT, DE, FL, GA, ID, KS, LA, MD, MA, MI, MS, MO, MT, NV, NJ, NY, NC, OK, OR, RI, SC, TX, UT, VA, WA, WY	BC, NS
5, 7	*Schoenoplectus acutus* (Muhl.)	AN	AK, AZ, AR, CA, CO, CT, DE, ID, IL, IN, IA, KS, KY, ME, MD, MA, MI, MN, MO, MT, NE, NV, NH, NJ, NM, NY, NC, ND, OH, OK, OR, PA, RI, SD, TX, UT, VT, VA, WA, WV, WI, WY	AB, BC, MB, NB, NS, NT, ON, PE, QC, SK, YT
**Typhaceae, Typhoideae**				
5, 7	*Typha latifolia* L.	–	AL, AK, AZ, AR, CA, CO, CT, DE, FL, GA, ID, IL, IN, IA, KS, KY, LA, ME, MD, MA, MI, MN, MS, MO, MT, NE, NV, NH, NJ, NM, NY, NC, ND, OH, OK, OR, PA, RI, SC, SD, TN, TX, UT, VT, VA, WA, WV, WI, WY	AB, BC, MB, NB, NL, NS, NT, ON, PE, QC, SK, YT
5, 7	*Typha angustifolia* L	–	AK, AR, CA, CO, CT, DE, ID, IL, IN, IA, KS, KY, LA, ME, MD, MA, MI, MN, MS, MO, MT, NE, NV, NH, NJ, NM, NY, NC, ND, OH, OK, OR, PA, RI, SC, SD, TN, VT, VA, WA, WV, WI, WY	BC, MB, NB, NS, ON, PE, QC, SK,
**Sparganiaceae**				
5, 7	*Sparganium americanum* Nutt.	–	AL, AR, CT, DE, FL, GA, IL, IN, IA, KS, KY, LA, ME, MD, MA, MI, MN, MS, MO, ND, NH, NJ, NY, NC, OH, OK, PA, RI, SC, TN, TX, VT, VA, WV, WI	MB, NB, NL, NS, ON, PE, QC
**Pontederiaceae**				
5	*Pontederia cordata* L.	–	AL, AR, CT, DE, FL, GA, IL, IN, IA, KS, KY, LA, ME, MD, MA, MI, MN, MS, MO, NH, NJ, NY, NC, OH, OK, OR, PA, RI, SC, TN, TX, VT, VA, WV, WI	NB, NS, ON, PE, QC,
			
**Juncaceae**				
5, 7	*Juncus effusus* L.	–	AK, AZ, AR, CA, CO, CT, DE, FL, GA, ID, IL, IN, IA, KS, KY, LA, ME, MD, MA, MI, MN, MS, MO, MT, NE, NV, NH, NJ, NM, NY, NC, ND, OH, OK, OR, PA, RI, SC, TN, TX, VT, VA, WA, WV, WI	BC, MB, NB, NS, NT, ON, PE, QC
			
**Iridaceae**				
5	*Iris versicolor* L.	–	DE, ID, IL, KY, ME, MD, MA, MI, MN, NH, NJ, NY, OH, PA, RI, VT, VA, WI	MB, NB, NL, NS, ON, PE, QC

^1^ TAG Test Plant Categories include: **1)** genetic types of target weed, **2)** species of the same (or closely related) genus, **3)** species in the same family as the target weed (**3a** plants in same sub-tribe; **3b** plants of other sub-tribes; **3c** plants in same subfamily other tribes; and **3d** plants in other subfamilies), **4)** threatened and endangered species in the same family, **5)** species in other families in the same order having similar characteristics as target plant, **6)** species in other orders that have some physiological, morphological or biochemical similarities to the target weed including environmentally and economically important species, **7)** any plant on which the biological control agent or its close relatives have been found or recorded to feed and/or reproduce.

Our two selected potential weed biocontrol agents *A. geminipuncta* and *A. neurica* are widespread in Europe, but neither moth is found where average temperatures exceed 15 °C [4]. However, in North America the host plant is currently distributed throughout most of the US, including regions of the Gulf Coast with a distinctly different climate. We tested whether southern climate conditions as they may exist in Florida or in the Mississippi River Delta would allow successful overwintering of eggs. To compare winter survival, we selected long-term winter temperature profiles of cities located within the native European range of the species and southern US locales (Table 2).

**Table 2:** Average day/night temperatures (°C) along the US Gulf Cost (Fort Pierce and New Orleans), typical climate conditions where *P. australis berlandieri* occurs, and in northern (Stockholm), and central Europe (Basel), both in the core distribution area of *A. geminipuncta* and *A. neurica* and of Rome, Italy at the southern edge of the moth׳s native range.

Temperature	Fort Pierce, Florida^a^		New Orleans, Louisiana^b^		Stockholm, Sweden^c^		Basel, Switzerland^d^		Rome, Italy^e^	
	Day	Night	Day	Night	Day	Night	Day	Night	Day	Night
January	22.3	11.1	16.7	8.3	1	-3	5	-1	12	3
February	23.3	12.4	18.3	10	1	-4	7	-1	14	3
March	24.9	14.2	21.7	12.8	4	-2	12	2	16	6
April	26.8	16.7	25	16.1	10	1	16	5	19	8
May	29.3	20.5	28.3	20	16	6	20	9	24	12
June	31.1	22.8	31.1	23.3	20	11	24	12	28	16
July	32.1	23.3	32.2	24.4	23	14	26	14	31	19
August	31.9	23.5	32.2	24.4	21	13	25	14	32	19
September	31.1	23.3	30	22.8	16	9	21	10	27	15
October	29	20.7	26.1	17.8	10	5	16	7	23	12
November	25.9	16.3	21.1	12.8	5	1	9	3	17	7
December	23.3	12.9	17.8	8.9	2	-2	6	0	13	4

Source of climate data:

a http://www.usclimatedata.com/climate/fort-pierce/florida/united-states/usfl0156/2017/1.

b http://www.usclimatedata.com/climate/new-orleans/louisiana/united-states/usla0338.

c https://www.timeanddate.com/weather/sweden/stockholm/climate.

d https://www.timeanddate.com/weather/switzerland/basel/climate.

e https://www.timeanddate.com/weather/italy/rome/climate.

## 2 Experimental design, materials and methods

Detailed life-histories of our two experimental organisms *A. geminipuncta* and *A. neurica* are well described [5]. Eggs overwinter under leaf sheaths with tightly synchronized larval emergence just as *P. australis* shoots begin to elongate in spring. Larvae feed internally in the soft, nutrient- rich tissues within *P. australis* shoots above the growing point. Larval development takes several weeks and mature larvae pupate in lower sections of stems. Adults fly in late July and August with *A. neurica* occurring about two weeks before *A. geminipuncta*.

### 2.1 Larval acceptance

We conducted our experiments on larval acceptance of different test plant species in quarantine at the University of Rhode Island (URI) and in Europe at CABI in Delémont, Switzerland (CABI). We either purchased or field collected test plant species and propagated them in common gardens at either location. We obtained eggs and larvae of *A. geminipuncta* and *A. neurica* from captive colonies maintained outdoors at CABI (source of the captive colony were adults and larvae originally collected near CABI). At URI we kept eggs in an incubator (4 °C) before bringing them to room temperature to stagger tests in the spring easing logistical and space constraints in quarantine, which enabled us to synchronize larval hatch and plant growth. At CABI we also tested a subset of crop and wetland plants available in Europe.

In all our different no-choice tests we used a fine paint brush to transfer neonate larvae onto cut shoots or potted test plants using 6 - 15 replicates per test plant species in gauze cages or variable sizes [6]. Since these tests needed to be conducted over many years, we simultaneously assessed validity of each test using *P. australis* shoots or plants as controls using identical replication. Depending on testing conditions, we assessed larval survival, recording feeding marks, entrance or exit holes, and presence of frass after 5–14 days. We only considered a particular sequence of tests valid if larvae attacked and survived on European or North American *P. australis* set up simultaneously as controls. Species that allowed larval feeding or survival are indicated with either AN (*A. neurica*) or AG (*A. geminipuncta*) in Table 1 and were advanced to more detailed tests, including female oviposition choice, or larval discrimination in multiple-choice tests and results are reported elsewhere [6].

### 2.2 Oviposition choice experiments

In our oviposition tests we progressed from single- to multiple-choice tests using cut shoots (Fig. 2A; [6]) or potted plants (Fig. 2C, [6]) of European or North American *P. australis* or native *P. australis americanus* in small (40 × 40 × 65 cm, Fig. 2 A; [6]) or large gauze cages (2 × 2 × 1.6 m; Fig. 2C; [6]). We conducted these tests over multiple years (2003–2005) in accordance with availability of plant material or adult moths. We released a single *A. geminipuncta* or *A. neurica* pair in each test using cut shoots, and exchanged shoots and recorded all eggs laid every two days. We released five mated *A. geminipuncta* or *A. neurica* pairs for each test using potted plants in late July or early August as adults became available. We examined each shoot and recorded the number of eggs laid on each plant in early September.

### 2.3 Egg survival under southern climates

The distribution of *P. australis berlandieri* in the U.S. is restricted to areas south of 35° latitude, while the two moths occur only north of 35° latitude in Europe [4]. To investigate the potential of the two moths to establish in climates where *P. australis berlandieri* currently occurs, we set up an egg overwintering experiment with *A. geminipuncta* and *A. neurica* at URI in October 2017. We set incubator (Percival I-36LL, Percival, Perry, Iowa, USA) conditions to photoperiod and fluctuating average day and night temperatures of Fort Pierce, Florida and Basel, Switzerland [6]. We reprogrammed incubator conditions twice a week to follow seasonal changes at Fort Pierce and Basel (Table 2). We started to check for larval emergence weekly starting in February 2018, until we terminated the experiment in May 2018 [6].

### 2.4 No-choice, single-choice and multiple-choice oviposition tests at CABI

In no-choice oviposition tests in 2003, females accepted all plants for oviposition but laid almost three times as many eggs on European *P. australis* compared to native *P. australis americanus* (Table 3). Females retained eggs until death rather than ovipositing them on apparently less suitable hosts, including native *P. australis americanus* (Häfliger, unpublished data). Under these no-choice conditions, both *A. donax* and *P. arundinacea* were also accepted for oviposition with egg numbers lowest for *A. donax* (Table 3). In the multiple-choice oviposition test using potted plants we found similar results with no oviposition on native *P. australis americanus*, *A. donax* and *P. arundinaceae* (Table 3). However, the total number of eggs laid by five females was only 61, and we found 10 eggs on *T. latifolia* (Table 3) questioning whether tests of this type can deliver reliable results. Tests with increased replication in 2004 confirmed the high (but this year not absolute) avoidance of native *P. australis americanus* by *A. geminipuncta*. When we provided females a choice between cut shoots of European *P. australis* and native *P. australis americanus*, we found eggs exclusively on European *P. australis* (Table 4). When we offered females cut shoots of European *P. australis*, North American *P. australis* and native *P. australis americanus*, the number of eggs laid on native *P. australis americanus* was substantially lower than the number of eggs on introduced North American *P. australis* (Table 4), but only slightly lower than on European *P. australis* (which was the only lineage attacked in single-choice tests in 2003; Table 3). In our field cage multiple-choice oviposition tests with potted plants, females showed strong discrimination between European *P. australis* and native *P. australis americanus* in some years, but not others (Table 4), differences we are unable to explain.

**Table 3:** Results of no-choice oviposition test with *A. geminipuncta* using cut shoots and multiple-choice tests using potted plants in 2003. Data are means ± SE of two females/test on cut shoots and cumulative oviposition of five pairs releases consecutively in the multiple-choice test.

Plant/Lineage	No-choice	Multiple choice
	No. of eggs/female	
	Cut shoots	Potted plants
European *P. australis*	80. 5 ± 26.5	51
Native *P. australis americanus*	32.0 ± 32.0	0
*Arundo donax*	9.5 ± 9.5	0
*Phalaris arundinacea*	54.5 ± 54.5	0
*Typha latifolia*	–	10

**Table 4:** Results of single- and multiple-choice oviposition test with *A. geminipuncta* and *A. neurica* using cut shoots or potted plants of three different *Phragmites* lineages from 2003 to 2005. Data are means ± SE of 2 (2003), 4 (2004) or 3 (2005) replicates/test.

	A*. geminipuncta*	*A. neurica*
Lineage	No. eggs	
**2003, Cut shoots**		
European *P. australis*	103.5 ± 4.5	–
Native *P. australis americanus*	0.0 ± 0.0	–
**2004, Cut shoots**		
European *P. australis*	17.3 ± 9.0	–
North American *P. australis*	45.0 ± 8.7	–
Native *P. australis americanus*	11.5 ± 3.9	–
**2005, Cut shoots**		
North American *P. australis*	29.8 ± 14.9	27.6 ± 10.6
Native *P. australis americanus*	28.8 ± 8.5	15.0 ± 6.5
**2004, Potted plants**		
European *P. australis*	14.1 ± 4.5	–
Native *P. australis americanus*	19.1 ± 8.4	–
**2005, Potted plants**		
North American *P. australis*	66.0 ± 3.59	166.5 ± 27.2
Native *P. australis americanus*	16.7 ± 14.2	144.8 ± 43.6

In 2005, when we focused on discrimination between the introduced and native North American *Phragmites* lineages, we found no difference in the number of eggs laid by *A. geminipuncta* on introduced *P. australis* vs. native *P. australis americanus* using cut shoots, but tests with *A. neurica* showed a distinct preference for the introduced lineage (Table 4). Interestingly we found the reverse pattern for the two species in our field cages in which *A. geminipuncta* showed a strong, although not absolute, preference for introduced *P. australis* over native *P. australis americanus*, while *A. neurica* females distributed their eggs evenly among different lineages (Table 4).
