# Supplementary material for: Complete host specificity test plant list and associated data to assess host specificity of Archanara geminipuncta and Archanara neurica, two potential biocontrol agents for invasive Phragmites australis in North America
Source: Data Brief. 2018 Jun 26;19:1755–64. doi: 10.1016/j.dib.2018.06.068 (PMC6141379; doi:10.1016/j.dib.2018.06.068)
Supplement: Supplementary file 1 — Supplementary material [file mmc1.docx]

Declaration of Interest: none

This work has not been submitted to any other journal for review and it has not been published previously
